# TRAF3: Guardian of T lymphocyte functions

**DOI:** 10.3389/fimmu.2023.1129251

**Published:** 2023-02-06

**Authors:** Emma L. Hornick, Gail A. Bishop

**Affiliations:** ^1^ Department of Microbiology and Immunology, The University of Iowa, Iowa City, IA, United States; ^2^ Department of Internal Medicine, The University of Iowa, Iowa City, IA, United States; ^3^ Research, Iowa City Veterans Affairs Medical Center, Iowa City, IA, United States

**Keywords:** T lymphocyte, signaling, TRAF3, T cell receptor, phosphatase

## Abstract

Tumor necrosis factor receptor (TNFR)-associated factor 3 (TRAF3) is an adapter protein with many context-specific functions. Early studies of lymphocyte TRAF3 hinted at TRAF3’s importance for T cell function, but elucidation of specific mechanisms was delayed by early lethality of globally TRAF3^-/-^ mice. Development of a conditional TRAF3-deficient mouse enabled important descriptive and mechanistic insights into how TRAF3 promotes optimal T cell function. Signaling through the T cell antigen receptor (TCR) fails to induce normal proliferation and survival in TRAF3*^-/-^* T cells, and TCR-activated cells *in vitro* and *in vivo* have deficient cytokine production. These defects can be traced to incorrect localization and function of negative regulatory phosphatases acting at different parts of the signaling cascade, as can dysregulated effector responses and memory T cell homeostasis *in vivo* and an enlarged regulatory T cell (Treg) compartment. The important regulatory activity of TRAF3 is also evident at members of the TNFR superfamily and cytokine receptors. Here, we review significant advances in mechanistic understanding of how TRAF3 regulates T cell differentiation and function, through modulation of signaling through the TCR, costimulatory receptors, and cytokine receptors. Finally, we briefly discuss the recent identification of families carrying single allele loss-of-function mutations in *TRAF3*, and compare the findings in their T cells with the T cell defects identified in mice whose T cells completely lack T cell TRAF3. Together, the body of work describing TRAF3-mediated regulation of T cell effector function and differentiation frame TRAF3 as an important modulator of T cell signal integration.

## 1 Introduction

**Figure 1:**
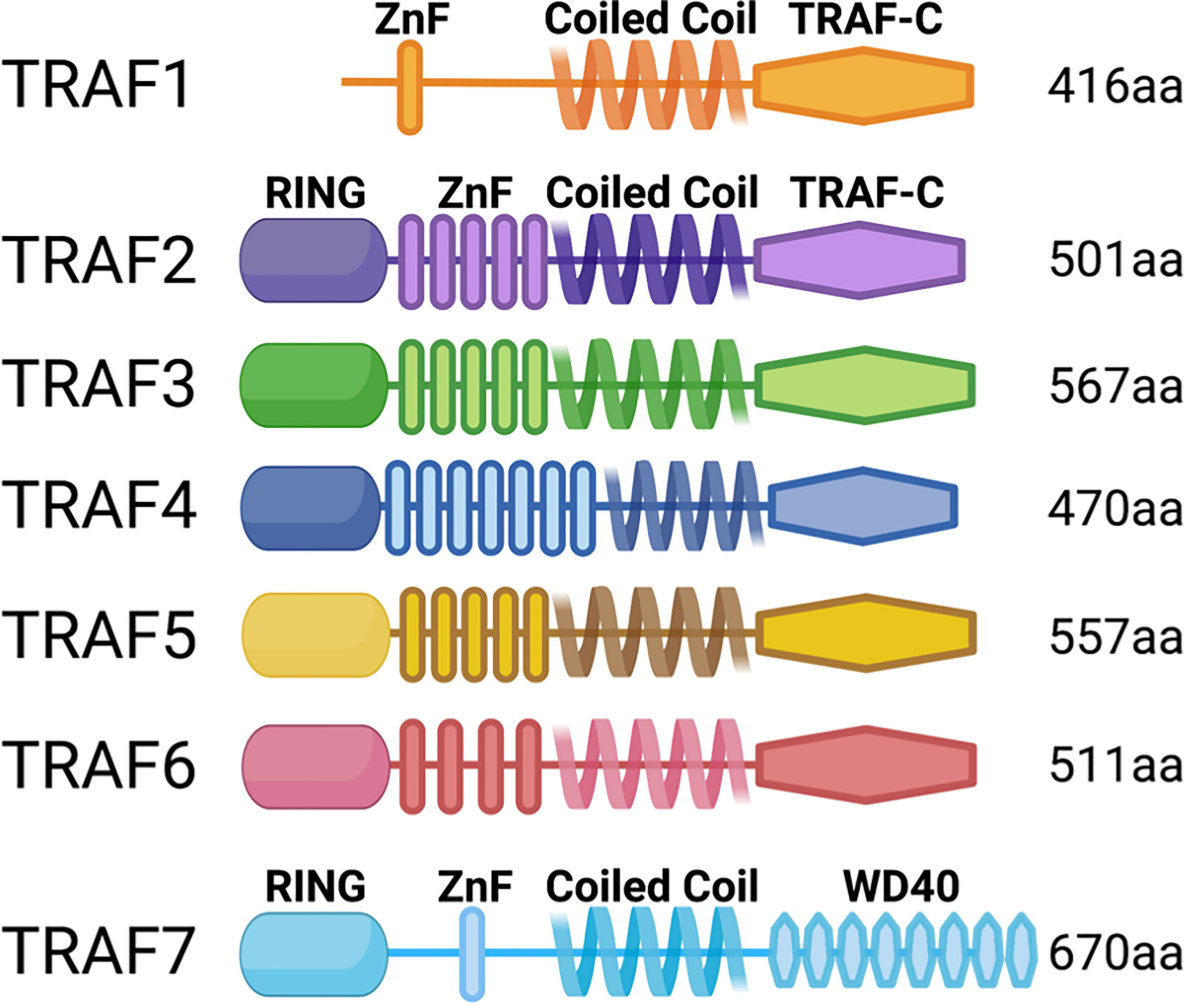
TRAF family member domain structures. TRAFs 2-6 are structurally very similar, but TRAFs 1 and 7 are distinct: TRAF1 lacks the RING domain, and TRAF7 lacks the TRAF-C domain and instead has C-terminal WD40 repeats.

The TRAF family of adaptor molecules regulates signals through many cytokine, antigen, and growth factor receptors (1). The seven TRAF family members share significant structural similarity, particularly TRAFs 2-6 (**Figure 1**). All TRAFs but TRAF1 have an N-terminal Really Interesting New Gene (RING) domain, which confers E3 ubiquitin ligase activity to some TRAFs. The TRAF discussed in detail below, TRAF3, has a RING domain but displays no enzymatic activity as part of its function in T cells. Following the RING domain is a variable number of zinc finger motifs and a C-terminal TRAF domain, which is crucial for protein-protein interactions. The N-terminal coiled-coil domain facilitates TRAF multimerization, and the highly conserved TRAF-C domain is important for other protein interactions (2). TRAFs 3 and 4 also have nuclear localization signals in the TRAF-C domain (3, 4).

TRAF3, ubiquitously-expressed in mammalian cells, is the only TRAF whose overexpression does not induce canonical NF-κB activation (5). Homozygous germline deletion of *Traf3* is lethal in mice shortly after birth (6). *Traf3^-/-^* pups are born indistinguishable from *Traf3^+/+^* littermates, but are visibly smaller by three days of age, and die within nine days. *Traf3^-/-^* pups exhibit progressive hypoglycemia and loss of immune cells over the first week of life, despite continued bone marrow hematopoiesis (6). This early lethality and the lack of conditional gene deletion technology in mice at that time prevented attribution of phenotypic features to defects in specific cell types lacking TRAF3. The first hint of an important role for TRAF3 in T cell function came from lethally irradiated wild-type mice reconstituted with *Traf3^-/-^* fetal liver cells, then vaccinated with T-dependent or T-independent antigens. The T-independent, but not T-dependent antibody response is intact, suggesting ineffective T cell help (6). Consistent with a T cell-intrinsic defect, *in vivo*-primed TRAF3*^-/-^* T cells do not proliferate as extensively as TRAF3*^+/+^* T cells following ex vivo antigen stimulation. These findings first implicated TRAF3 as an important regulator of T cell activation and effector function.

Subsequent development of a conditional TRAF3-deficient mouse confirmed the significance of T cell TRAF3 and facilitated more detailed characterization of TRAF3-deficient T cells (7, 8). TRAF3 regulates signals through the TCR, costimulatory receptors, and multiple cytokine receptors in T cells (9, 10). TRAF3 is also important for important for invariant Natural Killer T (iNKT) cell development through its regulation of the IL-15 receptor (IL-15R) and TCR (11). In this review, we discuss how TRAF3 acts as a guardian of T cell function and responsiveness for each of the three types of activating signals, ultimately facilitating robust and effective T cell responses.

## 2 Phenotype of mice lacking T cell TRAF3

*Traf3* deletion early in T cell development (*Lck*-Cre) causes a decrease in the number of mature peripheral T cells (12). In contrast, *Traf3* deletion at the CD4^+^CD8^+^ (*Cd4*-Cre, T-*Traf3^-/-^*) stage, or specifically in regulatory T cells (Treg, Treg-*Traf3^-/-^*) does not affect total numbers of mature T cells; the only exception is an approximately two-fold increase in thymic Treg in T-*Traf3^-/-^* mice, and a marked reduction in mature iNKT cells (11–13).

Closer investigation revealed alterations in naïve and effector/memory subsets. T-*Traf3^-/-^* and Treg-*Traf3^-/-^* mice have increased effector/memory CD44^+^CD4^+^ T cells and correspondingly decreased naïve (CD44^low/-^) CD4^+^ T cells compared to control mice (14). The CD44 expression profile of CD4^+^ T cells from T-*Traf3^-/-^* mice is also altered; there are approximately equal numbers of CD44^int^ and CD44^hi^ cells within the CD44^+^CD4^+^ T cell population in T-*Traf3^-/-^* mice, whereas most CD44^+^CD4^+^ T cells from control mice are CD44^hi^ (8, 14).

In contrast to CD4^+^ T cells, CD8^+^ T cells in T-*Traf3^-/-^* mice comprise normal numbers of naïve and effector memory cells, but reduced CD44^hi^CD62L^hi^ CD8^+^ T central memory (Tcm) cells. The reduction is due to impaired IL-15-mediated activation of Extracellular Signal-Related Kinase (ERK) and Signal Transducer and Activator of Transcription 5 (STAT5) in Tcm cells, which in turn impairs cell survival (14).

IL-15 is also a critical determinant of survival for iNKT cells, which are dramatically reduced in T-*Traf3^-/-^* mice compared to littermate controls (11). iNKT cell differentiation in T-*Traf3^-/-^* mice is blunted by diminished IL-15R-induced STAT5 activation and TCR-induced ERK activation. As a result, the levels of pro-survival molecule Bcl-X_L_ and the transcription factor T-bet in TRAF3^-/-^ iNKT cells are insufficient for cell expansion and survival (11).

*Listeria monocytogenes* (Lm) infection generates a robust T cell response in wild-type (WT) mice, required for successful bacterial clearance. T-*Traf3^-/-^* mice succumb to a low-dose Lm infection while TRAF3-sufficient littermates recover completely (8). In addition to increased mortality, T-*Traf3^-/-^* mice have delayed Lm clearance, fewer Lm-specific CD8^+^ T cells, and suboptimal T cell cytokine production following infection (5). T-*Traf3^-/-^* mice also fail to mount T-dependent antibody responses to immunization, consistent with the immunization studies in chimeric mice reconstituted with TRAF3-deficient fetal liver cells described above.

Confirming the importance of TRAF3 for T cell function—suppressive or supportive—Treg-*Traf3^-/-^* mice show increased germinal center formation, plasma cell differentiation, and elevated antibody titers following T-dependent immunization, due to the diminished differentiation and suppressive capacity of the follicular regulator T cell (Tfr) subset of TRAF3^-/-^ Treg cells (13).

## 3 *In vitro* activation of T-*Traf3^-/-^* mouse T cells

The findings above established TRAF3’s importance for robust T cell responses, but its role in individual signals in the complex environment of immunization or infection remained unclear. Examination of T cells activated *in vitro* (i.e., with a known set of stimuli) began to clarify how TRAF3 deficiency affects T cell activation.

TRAF3^-/-^ CD4^+^ T cells activated through CD3/TCR and CD28 fail to increase surface expression of early activation markers CD25 and CD69, proliferate, or survive as well as CD4^+^ T cells from littermate controls (8, 14). TRAF3^-/-^ CD4^+^ T cell cultures also contain less IL-2, IL-4, IL-17 and interferon gamma (IFNγ) than identically-treated control CD4^+^ T cells after TCR/CD28 stimulation (8, 14). PMA and ionomycin, which mimic downstream TCR signaling events through activation of Protein Kinase C and increasing intracellular Ca^2+^, induce more TRAF3^-/-^ CD4^+^ T cells than control CD4^+^ T cells to produce IFNγ and IL-10, with no difference in production of IL-2, TNF, or IL-17 (8, 14).

The TRAF3^-/-^ CD8^+^ T cell response to *in vitro* activation through either TCR/CD28 or PMA/ionomycin is distinct from that of TRAF3^-/-^ CD4^+^ T cells. Similar percentages of TRAF3*^-/-^* and control TCR/CD28-activated CD8^+^ T cells increase surface expression of CD69 and CD25, whereas IL-2, IFNγ, and TNF production is dramatically lower in TRAF3*^-/-^* CD8^+^ T cells (8, 14). Together, these findings emphasize the need for TRAF3 in TCR/CD28 signaling, and re-affirm the context-dependence of TRAF3 function.

## 4 TRAF3 in TCR signal transduction

TCR complexes of αβ T cells include the antigen-binding TCRα and β receptor chains, and CD3 subunit hetero- and homodimers, whose immunoreceptor tyrosine-based activation motifs (ITAMs) allow signal transduction. Upon TCR ligation, the CD4/CD8 coreceptor recruits the membrane-associated Lymphocyte-specific protein tyrosine Kinase (LCK) to the TCR complex to phosphorylate CD3 ITAMs and create a docking site for Zeta chain of TCR Associated Protein kinase 70 (ZAP70). Phosphorylation stabilizes ZAP70, which then phosphorylates the adaptor protein Linker for Activation of T cells (LAT), permitting formation of the LAT signalosome (15, 16). LAT signalosomes initiate signaling through the Ca^2+^-calcineurin-Nuclear Factor of Activated T cells (NFAT), mitogen-activated protein kinase (MAPK), and NF-κB pathways. Consistent with the impaired function of TRAF3*^-/-^* T cells, their activation through TCR/CD28 results in diminished LCK phosphorylation, leading to decreased phosphorylation of ZAP70 and LAT (8, 17). LAT signalosome-dependent activation of Phospholipase Cγ1 (PLCγ1), required for Ca^2+^ influx, and MAPK family member ERK are also decreased, while canonical NF-κB activation is unaffected. These findings establish TRAF3 as an important enhancer of early TCR/CD28 signaling (8).

**Figure 2:**
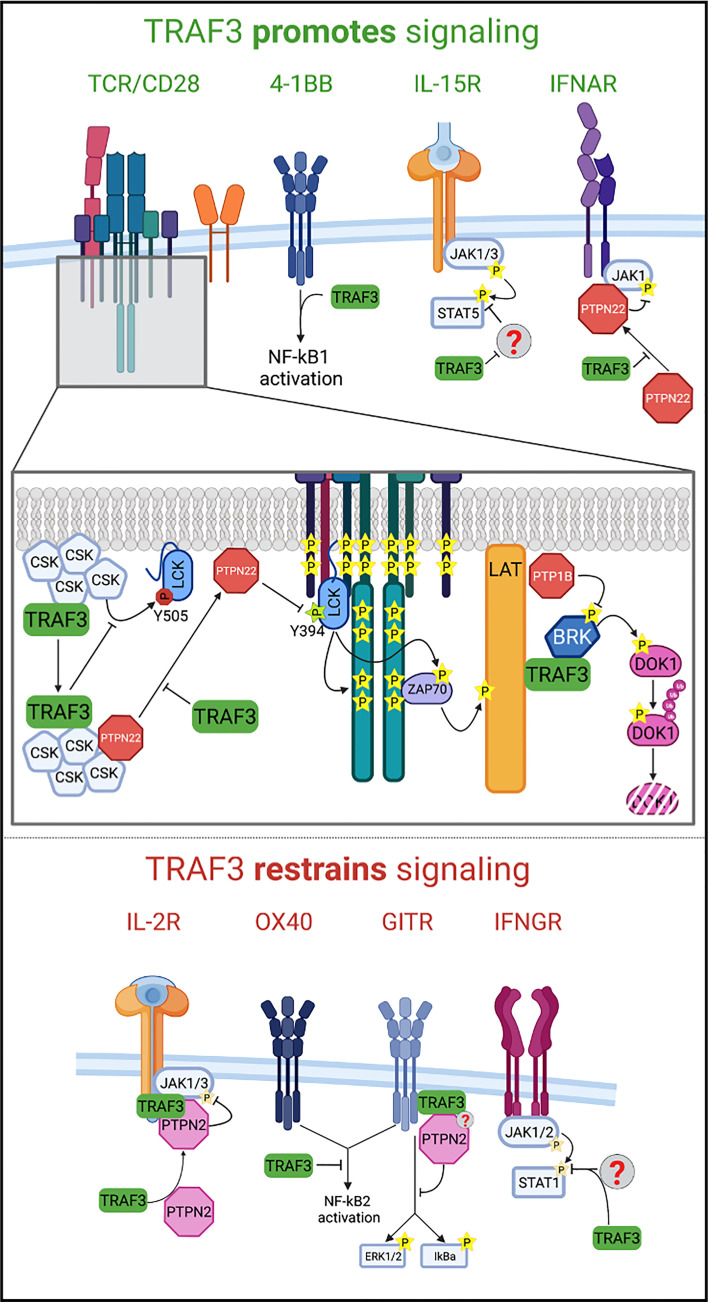
Mechanisms of TRAF3 regulation of T cell receptor signaling. Top: TRAF3 promotes signaling through the TCR/CD28, 4-1BB, IL-15R, and IFNAR by restraining negative regulators of signaling. Bottom: TRAF3 restrains signaling through the IL-2R in thymic Treg precursors and conventional CD4^+^ T cells, OX40, GITR, and IFNGR by mechanisms dependent on the phosphatase PTPN2 or unknown.

A shared theme among the mechanisms by which TRAF3 enhances TCR signaling is restraint of TCR inhibitors (**Figure 2**). The earliest demonstrable defect in TCR/CD28-stimulated TRAF3*^-/-^* T cells is reduced LCK activation (17). LCK activity is determined by the balance between levels of phosphorylation at activating (Y394) and inhibitory (Y505) residues. Negative regulation of LCK can occur through phosphorylation at Y505 by C-terminal Src Kinase (CSK), or through dephosphorylation of Y394 by Protein Tyrosine Phosphatase N22 (PTPN22). In WT T cells, TRAF3-CSK association increases in the cytoplasm and decreases in the plasma membrane following TCR/CD28 stimulation (17). In contrast, TRAF3*^-/-^* T cells show increased membrane-localized CSK, significantly increased activating phosphorylation of CSK, and slightly increased (inactive) pLCK^Y505^ with similar kinetics to the events in the WT T cells. Thus, TRAF3 enhances TCR signaling in part by rapidly sequestering CSK in the cytoplasm after TCR/CD28 stimulation, thereby preventing CSK from inactivating LCK at the membrane/TCR complex (**Figure 2**).

TRAF3^-/-^ T cells also show a reduction in pLCK^Y394^ following TCR/CD28 stimulation, starting 5-15 minutes after activation (17). Resting T cells maintain a pool of (active) pLCK^Y394^ to enable a rapid response to TCR ligation. The phosphatase PTPN22 is excluded from the plasma membrane through its cytoplasmic association with CSK. Following TCR/CD28 signaling, PTPN22 moves from the cytoplasm to the plasma membrane and dephosphorylates both pLCK^Y394^ and CD3 residues to curtail TCR signaling (17). In TRAF3*^-/-^* T cells (17), the cytoplasmic PTPN22-CSK association is reduced as PTPN22 becomes enriched in the plasma membrane, mirroring the kinetics of decreased pLCK^Y394^. Therefore, TRAF3 potentiates TCR signaling by facilitating CSK-PTPN22 association in the cytoplasm, preventing premature accumulation in the plasma membrane and inactivation of membrane-associated LCK (**Figure 2**).

Recruitment of TRAF3 to the TCR complex requires stimulation through both TCR and CD28 (8). The significance of this association and the requirement for both TCR and CD28 remained unclear until identification and experimental confirmation of a novel TRAF3 binding site in LAT. Interestingly, a TCR/CD28 stimulation-dependent association between TRAF3 and the LAT-associated negative regulator Downstream of Kinase 1 (DOK1) also occurs (18). In TCR/CD28-stimulated TRAF3*^-/-^* T cells, there is reduced inactive DOK1 (pDOK1^Y362^) and less polyubiquitination and proteasomal degradation of DOK1. DOK1 is a substrate of Breast tumor Kinase (BRK) (19), whose activating phosphorylation (pBRK^Y342^) and association with LAT are reduced in TCR/CD28-activated TRAF3*^-/-^* T cells compared to WT T cells. pBRK^Y342^ is dephosphorylated by Protein Tyrosine Phosphatase 1B (PTP1B), whose association with LAT is increased in TCR/CD28-stimulated TRAF3^-/-^ T cells. PTP1B inhibition rescues the defect in LCK activation in TRAF3*^-/-^* T cells (18). In summary, TRAF3 blocks recruitment of the negative regulator PTP1B to the LAT complex, which prevents the dephosphorylation-mediated inhibition of BRK, allowing BRK to phosphorylate and inactivate DOK1. This in turn prevents DOK1 from blunting LAT-mediated TCR signaling (**Figure 2**). In all the mechanisms described above, the absence of TRAF3 leads to suppression of TCR signaling through unchecked activity and inappropriate recruitment of negative regulators.

## 5 TRAF3 regulation of T cell costimulatory receptor signaling

Many TNFR superfamily members provide important pro-survival and pro-differentiation signals to T cells, and all bind at least one TRAF (1). TRAF3 associates with several of these family members. The biological significance of this interaction has to date been reported for 4-1BB/CD137, OX40/CD134, and GITR/CD357. TRAF3 is a dose-dependent enhancer of canonical NF-κB activation downstream of a CD19-specific Chimeric Antigen Receptor with a 4-1BB costimulatory domain (**Figure 2**). Increasing TRAF3 improves proliferation, persistence and cytokine production by the 4-1BB-CAR T cells (20). In contrast, TRAF3 negatively regulates non-canonical NF-κB activation downstream of OX40 (21).

GITR is one of a few constitutively-expressed costimulatory receptors on naïve T cells (22), permitting investigation of its regulation by TRAF3 without the confounding effect of suboptimal TCR signaling in TRAF3-deficient cells. TRAF3*^-/-^* T cells have higher GITR mRNA and protein than control T cells. In agreement with previous reports, this difference is not due to the increase in Treg cells in T-*Traf3^-/-^* mice, as GITR levels are similar on TRAF3-deficient and sufficient Treg cells (13, 23). Instead, the constitutive NF-κB2 activation present in all TRAF3-deficient cells is primarily responsible for upregulation of *Gitr* mRNA. Interestingly, however, the increase in GITR protein is not due entirely to increased mRNA; there is also reduced GITR protein turnover in the absence of TRAF3 (23). GITR signaling causes a TRAF-C-dependent recruitment of TRAF3 to the GITR cytoplasmic domain, and activates canonical NF-κB, MAPK and mTOR-AKT-S6 kinase pathways (23). Phosphorylation of IκBα, ERK, and S6 kinase are enhanced in TRAF3*^-/-^* T cells, but the enhanced S6 kinase activation is ERK-dependent rather than AKT-dependent. Interestingly, inhibition of Protein Tyrosine Phosphatase N2 (PTPN2) in GITR-stimulated TRAF3*^-/-^* T cells reduces MAPK and canonical NF-κB activation to WT T cell levels, but TRAF3 deficiency does not affect the PTPN2-GITR association (23). This work establishes a role for TRAF3 in costimulatory receptor signaling beyond CD28, and reinforces the context-dependence of TRAF3 activity in T cells, even among subsets within this single cell type.

## 6 TRAF3 regulation of cytokine receptor signaling

Cytokines are critical determinants of fate throughout T cell development and mature lifespan. In the thymus, T cells must demonstrate appropriate TCR signal strength to progress to the next stage of development, instructed in part by response to cytokines (24). In the periphery, successful receipt of signals one and two during T cell activation is a requirement for expansion and acquisition of effector functions (25). Signal three from cytokines further refines the differentiation path of a given T cell (26). TRAF3 regulates signals through cytokine receptors important for T cell development, differentiation, and effector function.

Enhanced IL-2R signaling drives increased thymic Treg differentiation in T-*Traf3^-/-^* mice (12). Mature Treg cells from T-*Traf3^-/-^* mice do not exhibit the same marked increase in IL-2R signaling, but conventional CD4^+^ T cells do, with important implications for the outcome of antigen-driven activation. IL-2R engagement induces activation of Janus kinases JAK1 and JAK3, whose phosphorylation and activation of STAT5 induce its nuclear translocation and initiation of *Foxp3* transcription (27). JAK1, JAK3, and STAT5 activation are enhanced in T-*Traf3^-/-^* thymic precursor Treg and conventional CD4^+^ T cells, and there is more STAT5 associated with target genes, including the high-affinity IL-2Rα chain (12). TRAF3 mediates negative regulation of this signaling pathway by recruiting PTPN2/TCPTP to the IL-2R complex, where it dephosphorylates JAKs to interfere with signaling. IFNγ receptor (IFNGR) signaling is also regulated by PTPN2 and is enhanced in TRAF3*^-/-^* CD4^+^ T cells (12).

TRAF3 also modulates signaling through T cell IFNAR. IFN I binding to IFNAR induces activation of JAK1 and TYK2, which then phosphorylate STATs. CD4^+^ T cells in T-*Traf3^-/-^* mice and a CD4^+^ TRAF3^-/-^ human T cell line have poor IFN I-induced JAK1 and STAT1 activation, leading to decreased ISG induction (28). The decreased JAK1 and STAT1 activation in TRAF3-deficient cells is likely due to abnormal association of the negative regulators PTPN2 and PTPN22 with the IFNAR complex, as minimal PTPN2 or PTPN22 associates with this complex upon stimulation in WT cells (28). Consistently, IFN I induces TRAF3 association with PTPN2 and PTPN22, potentially blocking phosphatase access to the IFNAR complex. Despite the robust association of both phosphatases with the IFNAR complex in TRAF3-deficient cells, only PTPN22 inhibition increases IFNAR-induced JAK1 and STAT1 activation to near-WT levels (28). Notably, TRAF3 deficiency does not affect the B cell response to IFN I, adding IFNAR signaling regulation to the list of cell type-dependent functions of TRAF3. Given the promiscuity of PTPN2 and PTPN22 in regulation of cytokine receptors, it is likely that TRAF3*^-/-^* T cells have other cytokine receptor signaling differences due to dysregulation or lack of regulation of these phosphatases, which makes them attractive targets for intervention in human patients with decreased TRAF3 abundance or functions.

## 7 Human disease and T cell TRAF3 deficiency

Until very recently, there were few reports of germline *TRAF3* deficiencies in humans, although somatic loss-of-function mutations are regularly documented in B cell malignancies (29–31). However, the recent identification of five unrelated families with mutations in one *TRAF3* allele that reduce total cellular TRAF3 by >50% has provided exciting new insight into the human disease-relevance of the studies described above (32). The clinical phenotype of patients with TRAF3 haploinsufficiency (*TRAF3* ^HI^) is marked by autoimmunity, immunodeficiency, and a predisposition to B cell malignancies, all of which are reminiscent of the immune cell-specific TRAF3 deficient mouse models. Similar to T-*Traf3^-/-^* mice, *TRAF3* ^HI^ patients have increased effector/memory CD4^+^ T cells and Treg cells, decreased naïve CD4^+^ T cells, decreased naïve CD8^+^ T cells, and defective IFNγ production upon *in vitro* stimulation (32). In contrast to the mouse model, overall T cell numbers are decreased, and Tfh are increased, though Foxp3 expression is lower in *TRAF3* ^HI^ patient Tfh, possibly reflecting decreased Tfr cells. Overall, the similarities to T-*Traf3^-/-^* mouse and human T cell line findings are striking and encouraging, particularly given the comparative heterogeneity among human subjects.

## 8 Discussion

TRAF3’s regulation of T cell signal transduction by antigen, costimulatory, and cytokine receptors has significant effects on T cell phenotype and function. TRAF3 exerts its effects by modulating recruitment of negative regulators, most often PTPs, to target receptors (2). It is interesting to consider how TRAF3 activity is constrained, given that it has targets that are shared among multiple signaling pathways. For example, only IFNAR-dependent STAT1 activation is affected by TRAF3 deficiency, although STAT5 is also activated by IFNAR signaling, and is regulated by TRAF3 in at least two other signaling cascades (11, 12). Determining how specificity of regulation is controlled, and whether the same TRAF3-mediated mechanism of regulation is applied to a given signaling intermediate regardless of the activating pathway could be very important for understanding how overlapping signaling pathways elicit distinct responses. A related question is the mechanism underlying TRAF3’s differential regulation of the same receptor (e.g., IFNAR) among different cell types. Further understanding of the effect of T cell TRAF3 deficiency on T cell function in the context of infection could also provide important information about signaling requirements in immune cell interactions, such as Tfh-GC B cell interactions. For example, TRAF3*^-/-^* Tfr cannot upregulate ICOS to the extent of WT Tfr (13), and ICOS signaling is critical for completion of Tfh differentiation and communication with GC B cells (33). Many interesting questions about T cell TRAF3 remain, but its promotion of TCR/CD28 signaling, restraint of thymic Treg development, and regulation of signaling through the IL-2, IL-15, IFN I and IFNγ receptors in conventional T cells establish TRAF3 as an important guardian of T cell functions.

## Author contributions

EH wrote the manuscript and prepared the figures. EH and GB edited and revised the manuscript and Figures. BioRender was used for figure preparation. All authors contributed to the article and approved the submitted version.
